# The Frequency, Preferences, and Determinants of Energy Drink Consumption Among Young Polish People After the Introduction of the Ban on Sales to Minors

**DOI:** 10.3390/nu17162689

**Published:** 2025-08-20

**Authors:** Patrycja Musz, Wiktoria Smorąg, Gabriela Ryś, Krzysztof Gargasz, Ewelina Polak-Szczybyło

**Affiliations:** 1 Student Scientific Club of Human Nutrition, Faculty of Health Sciences and Psychology, Collegium Medicum of Rzeszow University, ul. Warzywna 1a, 35-959 Rzeszow, Poland; patrycja.musz.10@gmail.com (P.M.); smorag.wiktoriaa@gmail.com (W.S.); rysgabrysia@interia.pl (G.R.); 2 Data Analysis Laboratory, Centre for Innovative Research in Medical and Natural Sciences, Faculty of Medicine, University of Rzeszow, ul. Warzywna 1a, 35-959 Rzeszow, Poland; kgargasz@edu.ur.pl; 3 Department of Dietetics, Faculty of Health Sciences and Psychology, Collegium Medicum of Rzeszow University, ul. Warzywna 1a, 35-959 Rzeszow, Poland

**Keywords:** energy drinks, caffeine, health risk, adolescents, safety, dietetics

## Abstract

**Background**: In Poland, the consumption of energy drinks among young people has changed significantly following the introduction of a ban on sales to minors. This regulatory measure was intended to address growing concerns about the health effects of high caffeine consumption among teenagers. The aim of the study was to assess the frequency, preferences, and determinants of energy drink consumption among Polish adolescents aged 15–17 years, following the introduction of the legal ban on the sale of energy drinks to minors. **Methods**: The study was conducted in Poland in 2024, following the introduction of a law prohibiting the sale of energy drinks to minors. The study group consisted of 999 high school students aged 15–17 who completed an anonymous, author-designed survey. Data were then collected and analyzed for descriptive statistics, and chi-square tests were used for categorical variables, Mann–Whitney U tests and Kruskal–Wallis tests for group comparisons, and Spearman’s rank correlation for ordinal data. **Results**: In total, 52% of respondents declared that they consume energy drinks, and 68% reported a reduced intake after the introduction of the regulations prohibiting sales to minors. These drinks are more frequently consumed by males. Additionally, age and frequency of consumption were positively correlated. The factors most often influencing the choice of energy drinks were price, taste, package size, caffeine content, composition, and recommendations from friends. **Conclusions**: Although a large percentage of minors still consume energy drinks, the new regulations have had an impact on reducing their intake.

## 1. Introduction

Energy drinks were introduced to the European market in the 1960s and began gaining popularity in Poland in the 1990s. Since then, their consumption has been steadily increasing. In 2006 alone, approximately 500 new energy drink brands were launched worldwide, leading to a 240% increase in sales in subsequent years. They are currently sold worldwide in more than 165 countries [1,2]. Energy drinks represent the fastest-growing segment of the beverage industry. Their composition and marketing strategies are constantly evolving in response to consumer preferences and emerging trends [2].

Originally, energy drinks were designed to enhance physical and mental performance. The effects offered by the producers of these drinks make them particularly popular among young people, students, athletes, and working adults. Extensive marketing campaigns targeted at youth—often featuring celebrities popular in this demographic—have contributed to their widespread consumption among increasingly younger individuals. It is now estimated that up to 50% of energy-drink consumers are children, adolescents, and young adults under the age of 25 [1,2].

Energy drinks commonly contain caffeine, sugars or sweeteners, herbal extracts, L-carnitine, taurine, and a range of vitamins. These components are mainly responsible for increasing energy in organisms, but they may also influence other physiological functions. Caffeine, a naturally occurring alkaloid, is absorbed in the digestive tract into the circulating blood and then distributed throughout the body [1,3]. The caffeine content in energy drinks varies by brand, ranging from 50 mg to as much as 500 mg in a 500 mL can. According to the European Food Safety Authority, the safe caffeine intake for children and adolescents is 3 mg per kilogram of body weight, not exceeding 100 mg per day [4]. An average can of energy drink (250 mL) contains approximately 80 mg of caffeine, which is similar to the amount of caffeine contained in 60 mL of espresso [5,6]. Among young people, there is a tendency to over-consume beverages containing high levels of caffeine [7]. Excessive consumption can negatively affect the cardiovascular system, hyperactivity, problems with length and quality of sleep, or gastrointestinal problems [8,9]. Youth who abuse beverages with a high dose of caffeine are also at greater risk of developing mental disorders such as anxiety disorders, depression, and educational problems. Studies suggest a potential link between excessive caffeine intake from this type of beverage and suicidal thoughts or behaviors [10]. Additionally, classic energy drinks contain high doses of sweeteners, which contribute to an increased risk of dental caries, obesity [11], type 2 diabetes, and other metabolic disorders associated with high-sugar diets [12,13]. Regular energy drink consumption has also been associated with a higher likelihood of developing addictive behaviors [14].

Until early 2024, the sale of energy drinks in Poland was not subject to legal restrictions. In the interest of the health of children and adolescents, a nationwide ban on the sale of energy drinks to individuals under 18 years of age came into force on 1 January 2024. This legislative measure aims to curb excessive caffeine consumption among minors. Under this regulation, the ban applies to beverages containing more than 150 mg/L of caffeine and/or taurine. Manufacturers who violate the law are subject to financial penalties imposed by the appropriate regulatory authorities [15,16]. In contrast, most EU countries have not implemented age-based sales restrictions for energy drinks. Currently, only Lithuania (since 2014) and Latvia (since 2016) prohibit the sale of these beverages to individuals under 18. In the United Kingdom, public consultations on potential regulation are still ongoing [13]. In the United States, the Food and Drug Administration (FDA) has not introduced specific laws regarding the sale or labeling of energy drinks, which are regulated under general food and beverage standards. However, the American Beverage Association (ABA) has published voluntary guidelines for the labeling and marketing of energy drinks [17].

The aim of this study was to assess the prevalence, patterns, and determinants of energy drink consumption among Polish adolescents aged 15–17 years in the context of newly implemented national legislation banning the sale of energy drinks to minors. The study also aimed to evaluate young people’s awareness of the potential health risks associated with energy drink consumption and determine whether the legal restriction influenced their consumption behaviors.

To our knowledge, this is the first study in Poland following the implementation of the regulations discussed above for this age group. This study fills a gap in knowledge on this topic that has been conducted on the adult population or in other countries [18,19].

## 2. Materials and Methods

### 2.1. Study Design

The project utilized data collected from high school students aged 15 to 17. The study was conducted between 10 March and 14 April 2024. Paper-based surveys were administered in high schools following prior consent from the school administration and the scheduling of appropriate dates. Individuals under the age of 15 and those who did not provide consent were excluded from the study. For subsequent statistical analysis, the collected data were entered into a password-protected Excel file. The original paper questionnaires were securely stored in a location accessible only to the research team. Blinded data were made publicly available in an open-access repository.

### 2.2. Data Collection Tool

The survey consisted of 11 questions regarding sex, age, place of residence, frequency, and typical volume (e.g., 250 mL, 355 mL, or 500 mL) of energy drinks consumed, as well as factors influencing the decision to consume and purchase a specific energy drink. A significant element of the survey also included questions regarding changes in the consumption of this type of beverage in relation to the introduction of the ban on its sales. The questions were designed by the authors to address the research questions related to the study’s aim. The questionnaire was not tested for validity or reliability. The survey featured closed-ended questions, with some allowing for multiple responses. The original questionnaires in Polish, along with their English translations (Supplementary File S1), are included in Supplementary Materials.

### 2.3. Ethics

Data were collected as part of a research project approved by the University Bioethics Committee of the University of Rzeszów (resolution no. 3/11/2023). Participation in the study was anonymous and entirely voluntary, and the study itself did not constitute a medical experiment. Informed consent to participate was obtained directly from students aged ≥15 years, in accordance with national ethical standards for non-invasive, anonymous survey research involving minors. Permission to conduct the study was also granted by the principals of the secondary schools in which recruitment took place. The purpose, scope, and procedures of the study were explained to the participants, who were informed that the questionnaire did not include sensitive data, that their responses could not be identified, and that they had the right to withdraw from the study at any time without giving a reason and without consequences. Parents/legal guardians received written information about the project, including its purpose, scope, the absence of sensitive data collection, and the voluntary nature of their children’s participation (Supplementary File S2). Given the anonymous nature of the study, the lack of interference with the educational process and the absence of sensitive data collection, written consent from parents/guardians was not obtained; it was assumed that the students’ conscious completion of the questionnaire, after prior information had been provided to them and their parents, constituted informed consent to participate in the study.

### 2.4. Statistical Analysis

The collected data were analyzed using Statistica software (StatSoft Polska, version 13.1). Descriptive statistics were used to present the distribution of responses, including the number of observations (*n*) and percentages (%). The Pearson’s chi-square test (Chi^2^) was used to assess the relationships between categorical variables, such as age, sex, and place of residence, in relation to energy drink consumption frequency and reasons for use. For comparisons between two independent groups (e.g., sex differences), the non-parametric Mann–Whitney U test was applied. To compare more than two groups (e.g., place of residence), the Kruskal–Wallis test was used. The strength and direction of associations between ordinal variables (e.g., age and frequency of consumption) were assessed using Spearman’s rank correlation coefficient. Statistical significance was established at a level of *p* < 0.05.

### 2.5. Study Group

**Figure 1:**
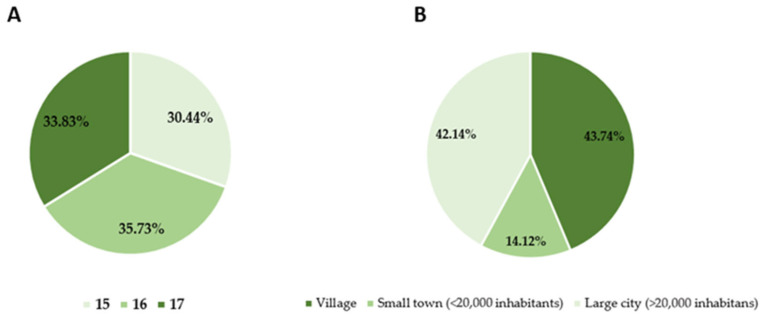
(**A**) Age and (**B**) place of residence of respondents.

The study group consisted of 999 minors. 582 women (58.26%) and 417 men (41.74%) participated in the study. The participants were students aged 15–17 from high schools in the Podkarpackie region. The place of residence varied, but the majority of the respondents—562 (56.26%)—came from the city and 437 (43.74%) from the countryside (Figure 1).

## 3. Results

Among the 999 respondents, 47.45% reported that they did not consume energy drinks, while only 7.71% consumed them more than once a week. It was noticed that the highest frequency of consumption was observed among 17-year-olds, who drank energy drinks several times a day more often than younger participants. In contrast, 15-year-olds were the most likely to report not consuming energy drinks at all (Table 1). Analysis confirms a statistically significant increase in the frequency of consumption with age (Chi^2^ NW test, *p* = 0.01; Spearman correlation R = 0.10, *p* < 0.05).

In addition to age, sex also played a significant role in the frequency of energy drink consumption. Females more frequently reported that they did not consume energy drinks at all (26.03%) compared to males (21.42%) and were also less likely to consume them several times a day (1.5% vs. 2%). In the case of other consumption frequencies, they occurred more often in females (Table 2). Statistical analysis shows that females were more likely to report consuming energy drinks in general (Chi^2^ NW test, *p* = 0.04), although there was no significant difference in consumption frequency between sexes (Mann–Whitney U test, *p* = 0.4). Regarding portion size, females more frequently chose smaller volumes (250 mL and 355 mL), whereas males preferred larger 500 mL cans (Chi^2^ NW test, *p* < 0.00001). In addition to age, sex also played a significant role in the frequency of energy drink consumption.

Place of residence was not associated with the declaration of energy drink consumption (Chi^2^ NW test, *p* = 0.7) and had no significant effect on the frequency of consumption (Kruskal–Wallis test, *p* = 0.7). It also did not influence the volume of energy drinks consumed (Chi^2^ NW test, *p* = 0.5) (Table 3).

The reasons why individuals under the age of 18 consume energy drinks were analyzed, with participants allowed to select multiple answers (Table 4). Taste was unanimously indicated as the primary reason for consumption, followed by using energy drinks as support during physical activity. The least common reason (1.2%) was peer influence or fashion (e.g., “because everyone drinks”). Analyzing the reasons for energy drinks by age and sex, age was found to be associated with consuming energy drinks when tired. Females were also more likely to consume these types of drinks to boost energy, while males were more likely to drink them in social situations and when tired. Place of residence was not significantly associated with the reasons for consumption.

**Figure 2:**
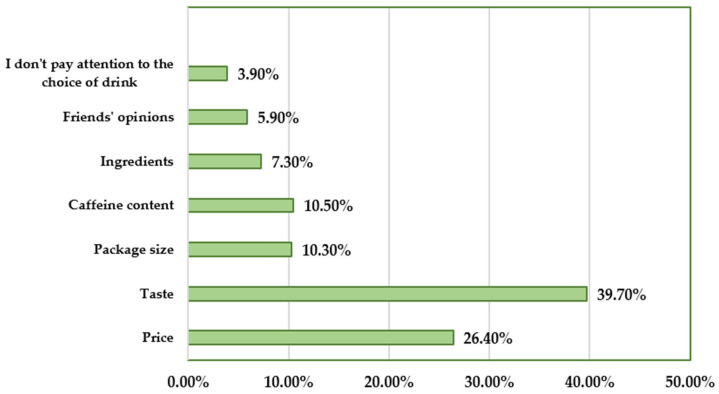
Important factors when purchasing energy drinks.

The reasons for choosing a specific type of product and the frequency of their declarations by respondents in the survey are presented in Figure 2. The most common reason was taste (*n* = 397, 39.7%), while the least common was the opinion of friends (*n* = 59, 5.9%). It should be noted that only 39 (3.9%) respondents do not pay attention to the type of energy drink they choose.

Statistical analysis does not reveal any association between place of residence and reasons for product choice. Regarding sex, females were more likely to select energy drinks based on taste and friends’ opinions. Additionally, with increasing age, taste became a more frequently reported factor influencing product selection (Table 5).

**Figure 3:**
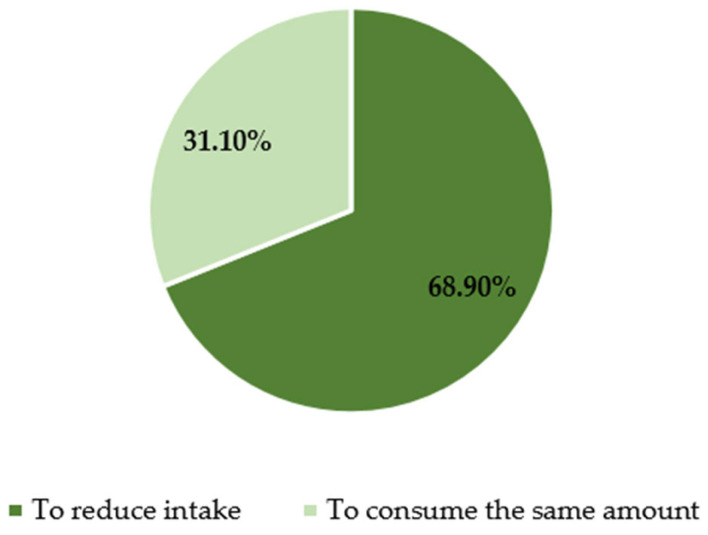
The trend of energy drink consumption over time.

In addition, minors were asked about their attitudes toward the new regulations. Among the 525 respondents who declared that they consumed energy drinks, 360 (68.9%) reported a reduction in consumption after 1 January 2024, while the remaining 31.1% continued to consume them at the same level (Figure 3). No significant differences were observed in these declarations with respect to sex, age, or place of residence.

## 4. Discussion

On 1 January 2024, a ban on the sale of energy drinks to children and adolescents under 18 years of age came into force in Poland. According to the regulations introduced, the ban covers beverages that contain more than 150 mg of caffeine or taurine, excluding substances that occur naturally in them [5]. Analyses conducted by the European Food Safety Authority indicate that, in recent years, energy drinks have been most commonly consumed by people aged 10 to 17, highlighting the emergence of this issue among young adolescents [20].

In our study, 52.5% of people under 18 years of age declared that they still consumed energy drinks, while nearly 70% of them declared that they reduced their consumption after the change in regulations in January 2024. These findings raise concerns about the effectiveness and enforcement of the amended legislation, which was intended to improve public health outcomes among adolescents. Another study conducted between 2012 and 2013 among Polish adolescents found that nearly two-thirds of surveyed high school students admitted to consuming energy drinks. Although our own study was carried out at a later time, the comparison of findings from both studies confirms a significant and persistent interest in such beverages among Polish youth [21]. Different results were observed in a study conducted among adolescents in Spain. According to the 2013 EFSA report, nearly 70% of respondents aged 10–18 consumed energy drinks, whereas the 2023 report indicated a decline to 47.7%, suggesting a downward trend. Although Spain has not established an official definition of energy drinks, some regions have introduced specific labeling requirements and a dedicated tax on such products. Additionally, a draft law has been proposed to prohibit the sale of caffeine-containing beverages to individuals under the age of 18 and to restrict their availability at events intended for children and adolescents [22]. An analysis of energy drink consumption across European countries indicates that the highest consumption rates are observed in Poland, the Czech Republic, and Hungary [18]. Numerous studies have demonstrated that the frequency of consuming such beverages increases with age and is generally higher among males [21,23]. Similar findings were reported in a study conducted in Spain [22]. In contrast, a study by Teijeiro et al., which examined the frequency of energy drink consumption among Spanish adolescents aged 14–18, showed an increase in consumption in both sexes, with a particularly pronounced rise among females [24]. In the present study, however, no significant differences in energy drink consumption between genders were observed. Discrepancies among the cited studies may stem from demographic differences, as well as from an unequal number of participants and gender imbalance within the analyzed samples. An increasing number of studies available in the literature indicate a high frequency of energy drink consumption, with many respondents reporting intake several times a day, a trend also confirmed by the authors’ own research [18,21]. In a study by Cisińska (2017), as many as 1 in 10 underage students declared daily consumption of energy drinks [25]. Against this background, data from international studies appear particularly noteworthy. Among Korean adolescents, regular consumption of energy drinks was significantly more prevalent compared to observations reported in European studies [26]. A study conducted among physically active adolescents in Poland found no association between place of residence and the frequency of energy drink consumption [27]. Similar results were obtained in the authors’ own research, which also did not reveal a significant correlation. In contrast, a study conducted among Norwegian adolescents indicated a higher prevalence of energy drink consumption among teenagers living in rural areas compared to their urban counterparts [28]. The impact of place of residence on energy drink consumption appears to be limited. Although some studies highlight differences between urban and rural youth, the overall evidence suggests that residence is not a decisive factor. This is primarily due to the widespread availability of energy drinks in both large urban centers and smaller towns.

In our study, the main factors influencing the purchase of energy drinks included taste, price, support for physical activity, fatigue reduction, advertising, and the company of friends. Numerous other studies have also highlighted peer pressure, easy availability in many grocery stores, and improved concentration during studying as equally important determinants [1,29]. Our study found that a common reason for consuming energy drinks was daytime fatigue. Similar findings have been reported by other researchers, who observed that shorter sleep duration, high levels of physical activity during the day, and prolonged screen time—particularly watching television—were associated with increased consumption of sugar-sweetened beverages and energy drinks [29,30,31]. A study by Granda et al. indicated a significant increase in energy drink consumption with age. Additionally, it examined the motives for consuming such beverages in relation to the gender of the respondents. Females were more likely to consume high-caffeine drinks to enhance concentration while studying, whereas males more frequently consumed energy drinks during physical activity [27]. Similar trends were observed in the authors’ own study, which also demonstrated an age-related increase in caffeine intake. The most commonly reported motivation among both female and male respondents was the desire to boost energy levels throughout the day. Furthermore, male participants identified social gatherings as a significant factor influencing their consumption of energy drinks.

The studies referenced in this article highlight a consistent upward trend in the consumption of energy drinks, both in Europe and globally, particularly among the youngest age groups. Such patterns of use may lead to excessive caffeine intake, which, over time, can result in more serious adverse health effects. Such behaviors may ultimately lead to caffeine overdose and, over time, contribute to the development of more severe adverse effects. These may include, among others, headaches, seizures, chest pain, sleep disturbances, cardiac arrhythmias, gastrointestinal issues, hyperactivity, and anxiety disorders [18]. In a study conducted by Nowak et al., nearly one-third of underage individuals aged 17 reported that they occasionally mix energy drinks with alcohol, which may further exacerbate the adverse side effects associated with excessive consumption of such products [21]. Analyzing a study by Fariz et al., which determined the relationship between the consumption of caffeinated beverages and poor mental and physical condition, as well as unhealthy dietary and lifestyle behaviors, we can conclude that people who consume energy drinks more often have worse health habits [29].

The level of energy drink consumption in Poland remains comparable to, or even exceeds, that observed in countries that have not implemented similar regulatory measures. This may suggest the limited effectiveness of legal provisions when not accompanied by a robust system of enforcement and youth-oriented education. In light of these findings, it becomes evident that legislation alone is insufficient to effectively restrict adolescents’ access to energy drinks. A comprehensive approach is required that integrates legal regulations with educational initiatives, preventive strategies, and effective enforcement mechanisms. Without such integration, there is a significant risk that regulatory measures will serve merely as symbolic acts, failing to achieve the intended public health outcomes.

## 5. Limitations

This study has several limitations that should be considered. First, data were collected exclusively in the Podkarpackie region, which may limit the generalizability of the results to other regions of Poland or other cultural and socioeconomic contexts. Second, the use of self-report questionnaires may have introduced recall or social desirability bias, particularly in responses regarding health behaviors and the impact of legal regulations. Furthermore, the questionnaire was designed by the authors and has not been tested for validity or reliability. Third, the cross-sectional design does not allow for the establishment of causal relationships between the variables studied. Further research using nationally representative samples and longitudinal methods is recommended to verify and extend these findings.

## 6. Conclusions

This study demonstrates that, despite the legal restriction on energy drink sales to minors introduced in Poland in January 2024, more than half of adolescents aged 15–17 still report consuming these beverages. However, the majority of consumers declared a reduction in intake following the regulatory change, suggesting its partial effectiveness. The frequency of consumption was shown to increase with age, though no significant differences were found between sexes in terms of frequency. Notably, females more often selected smaller package sizes.

Taste and the perceived support for physical activity emerged as the most common reasons for energy drink consumption, while peer influence and fashion played only a minor role. Although sex and age influenced specific motivations for consumption, place of residence did not significantly affect frequency, volume, or reasons for use.

The findings highlight the persistent popularity of energy drinks among adolescents and underscore the need for continued public health efforts, including education on caffeine-related health risks. The results also emphasize the importance of tailoring preventive strategies to specific demographic subgroups, considering age- and sex-related differences in motivation and behavior. This study contributes to the limited body of evidence assessing the early impact of legislative measures on youth consumption patterns and provides a foundation for further evaluation and policy refinement.

## Figures and Tables

**Table 1:** Frequency of energy drink consumption by age.

Frequency of Energy Drink Consumption	Age [Years]			Total
	15	16	17	
Does not consume	165 (16.52%)	165 (16.52%)	144 (14.41%)	474 (47.45%)
Very rarely	87 (8.71%)	131 (13.11%)	108 (10.81%)	326 (32.63%)
Less than once a week	18 (1.80%)	18 (1.80%)	26 (2.60%)	62 (6.21%)
More than once a week	15 (1.50%)	32 (3.20%)	30 (3.00%)	77 (7.71%)
Once a day	11 (1.10%)	4 (0.40%)	10 (1.00%)	25 (2.50%)
Several times a day	8 (0.80%)	7 (0.70%)	20 (2.00%)	35 (3.50%)
Total	304 (30.43%)	357 (35.74%)	338 (33.83%)	999 (100%)

%—percentage.

**Table 2:** Frequency of energy drink consumption by sex.

Frequency of Consumption	Sex		Total
	Females	Males	
Does not consume	260 (26.03%)	214 (21.42%)	474 (47.45%)
Very rarely	213 (21.32%)	113 (11.31%)	326 (32.63%)
Less than once a week	37 (3.70%)	25 (2.50%)	62 (6.21%)
More than once a week	44 (4.40%)	33 (3.30%)	77 (7.71%)
Once a day	13 (1.30%)	12 (1.20%)	25 (2.50%)
Several times a day	15 (1.50%)	20 (2.00%)	35 (3.50%)
Total	582 (58.26%)	417 (41.74%)	999 (100%)

%—percentage.

**Table 3:** Frequency of energy drink consumption by place of residence.

Frequency of Energy Drink Consumption	Place of Residence			Total
	Village	Small Town (Less Than 20 Thousand Inhabitants)	Large City (Over 20 Thousand Inhabitants)	
Does not consume	203 (20.32%)	65 (6.51%)	206 (20.62%)	474 (47.45%)
Very rarely	154 (15.42%)	42 (4.20%)	130 (13.01%)	326 (32.63%)
Less than once a week	34 (3.40%)	8 (0.80%)	20 (2.00%)	62 (6.21%)
More than once a week	29 (2.90%)	15 (1.50%)	33 (3.30%)	77 (7.71%)
Once a day	7 (0.70%)	3 (0.30%)	15 (1.50%)	25 (2.50%)
Several times a day	10 (1.00%)	8 (0.80%)	17 (1.70%)	35 (3.50%)
Total	437 (43.74%)	141 (14.11%)	421 (42.14%)	999 (100%)

%—percentage.

**Table 4:** Analysis of the reasons for consuming “energy drinks” in relation to sex and age.

Reasons for Consuming Energy Drinks	*n* (%)	Sex	Age
Because of the taste	999 (100)	No	No
As a support for physical activity	999 (100)	No	No
To increase energy	224 (22.4)	Females (Chi^2^ *p* = 0.00001)	No
When very tired	226 (22.6)	Males (Chi^2^ *p* = 0.00002)	Tendency grows with age (Chi^2^ *p* = 0.01)
Because of advertisements and the presence of celebrities in them	14 (1.4)	No	No
For company with friends	50 (5)	Males (Chi^2^ *p* = 0.007)	No
Because of fashion	12 (1.2)	No	No

*n*—number of observations; %—percentage; *p*—*p*-value (statistical significance); Chi^2^—Pearson’s chi-square test.

**Table 5:** Analysis of the reasons for product choice when purchasing “energy drinks” in relation to sex and age.

Question: What Are the Reasons for Purchasing a Particular Type of Energy Drink?	*n* (%)	Sex	Age
Taste	397 (39.7)	females (Chi^2^ *p* = 0.01)	tendency grows with age (Chi^2^ *p* = 0.0006)
Price	264 (26.4)	no	no
Caffeine content	105 (10.5)	no	no
Package size	103 (10.3)	no	no
Ingredients	73 (7.3)	no	no
Friends’ opinions	59 (5.9)	Females (Chi^2^ *p* = 0.01)	no
I don’t pay attention to the choice of drink	39 (3.9)	no	no

*n*—number of observations; %—percentage; *p*—*p*-value (statistical significance); Chi^2^—Pearson’s chi-square test.

## Data Availability

The data are available in the repository of the University of Rzeszów, ID: https://rdb.ur.edu.pl/handle/item/72 (accessed on 1 July 2025).

## Supplementary Materials

The following supporting information can be downloaded at: https://www.mdpi.com/article/10.3390/nu17162689/s1, Supplementary File S1: Survey in Polish and English; Supplementary File S2: Information for Parent/Legal Guardian in Polish and English.

## Abbreviations

The following abbreviations are used in this manuscript:

EU	European Union
FDA	Food and Drug Administration
ABA	American Beverage Association
UK	The United Kingdom
USA	The United States of America

## References

[1] Nadeem I.M., Shanmugaraj A., Sakha S., Horner N.S., Ayeni O.R., Khan M. (2021). Energy Drinks and Their Adverse Health Effects: A Systematic Review and Meta-analysis. Sports Health.

[2] Pawlas K., Hołojda P., Brust K. (2017). The evaluation of energy drinks consumption and their influence on people’s health on the basis of opinions provided by students of Wroclaw universities. Med. Śr..

[3] Costantino A., Maiese A., Lazzari J., Casula C., Turillazzi E., Frati P., Fineschi V. (2023). The Dark Side of Energy Drinks: A Comprehensive Review of Their Impact on the Human Body. Nutrients.

[4] EFSA European Food Safety Authority. https://www.efsa.europa.eu/en/topics/topic/caffeine?.

[5] https://www.gov.pl/web/psse-mysliborz/1-stycznia---zakaz-sprzedazy-energetykow-nieletnim.

[6] USDA FoodData Central. https://fdc.nal.usda.gov/.

[7] Ajibo C., Van Griethuysen A., Visram S., Lake A.A. (2024). Consumption of energy drinks by children and young people: A systematic review examining evidence of physical effects and consumer attitudes. Public Health.

[8] Rodak K., Kokot I., Kratz E.M. (2021). Caffeine as a Factor Influencing the Functioning of the Human Body—Friend or Foe?. Nutrients.

[9] Halldorsson T.I., Kristjansson A.L., Thorisdottir I., Oddsdóttir C., Sveinbjörnsson J., Benediktsson R., Sigfusdottir I.D., Jörundsdóttir H., Gunnlaugsdottir H. (2021). Caffeine exposure from beverages and its association with self-reported sleep duration and quality in a large sample of Icelandic adolescents. Food Chem. Toxicol..

[10] Low C.E., Chew N.S.M., Loke S., Tan J.Y., Phee S., Lee A.R.Y.B., Ho C.S.H. (2025). Association of Coffee and Energy Drink Intake with Suicide Attempts and Suicide Ideation: A Systematic Review and Meta-Analysis. Nutrients.

[11] López-Rodríguez G., Galván M., Galván-Valencia O., Gómez-Castillo J. (2025). Gain of Body Fat and Intake of Energy in Rats with Low Dose of Caloric and Non-Caloric Sweeteners Used in Reformulation Beverage in Mexico. Beverages.

[12] Ostalecka K., Folcik J., Augustyn K., Kukulińska W., Makowicz D., Dziubaszewska R. (2024). Impact of Energy Drinks on the Health of People from the Podkarpackie Voivode, Ship.

[13] Sanchis-Gomar F., Lavie C.J., Lippi G. (2024). Strict regulations on energy drinks to protect Minors’ health in Europe—It is never too late to set things right at home. Prev. Med..

[14] Benkert R., Abel T. (2020). Heavy energy drink consumption is associated with risky substance use in young Swiss men. Swiss Med. Wkly..

[15] (2019). Resolution No. 16/2019 of the Team for Dietary Supplements Concerning the Issuance of an Opinion on the Maximum Amount of Caffeine in the Recommended Daily Portion of Dietary Supplements. https://www.gov.pl/web/zdrowie/uchwaly-zespolu-do-spraw-suplementow-diety.

[16] (2023). Act of 14 April 2023 Amending the Act on Public Health and Certain Other Acts (Ustawa z dnia 14 Kwietnia 2023 r. o Zmianie Ustawy o Zdrowiu Publicznym Oraz Niektórych Innych Ustaw). https://isap.sejm.gov.pl/isap.nsf/DocDetails.xsp?id=WDU20230001231.

[17] https://www.americanbeverage.org/education-resources/policies-research/.

[18] Mularczyk-Tomczewska P., Gujski M., Koweszko T., Szulc A., Silczuk A. (2025). Regulatory Efforts and Health Implications of Energy Drink Consumption by Minors in Poland. Med. Sci. Monit. Int. Med. J. Exp. Clin. Res..

[19] Mularczyk-Tomczewska P., Lewandowska A., Kamińska A., Gałecka M., Atroszko P.A., Baran T., Koweszko T., Silczuk A. (2025). Patterns of Energy Drink Use, Risk Perception, and Regulatory Attitudes in the Adult Polish Population: Results of a Cross-Sectional Survey. Nutrients.

[20] Sorkin B.C., Coates P.M. (2014). Caffeine-containing energy drinks: Beginning to address the gaps in what we know. Adv. Nutr..

[21] Nowak D., Jasionowski A. (2015). Analysis of the Consumption of Caffeinated Energy Drinks among Polish Adolescents. Int. J. Environ. Res. Public Health.

[22] Leis R. (2025). Stimulating drinks: Neither energy drinks nor for teens. An. Pediatría.

[23] Vogel C., Shaw S., Strömmer S., Crozier S., Jenner S., Cooper C., Baird J., Inskip H., Barker M. (2022). Inequalities in energy drink consumption among UK adolescents: A mixed-methods study. Public Health Nutr..

[24] Teijeiro A., Pérez-Ríos M., García G., Martin-Gisbert L., Candal-Pedreira C., Rey-Brandariz J., Guerra-Tort C., Varela-Lema L., Mourino N. (2025). Consumption of energy drinks among youth in Spain: Trends and characteristics. Eur. J. Pediatr..

[25] Cisińska A. (2017). Eating habits of Lodz junior high school students. Pielęgniarstwo Polskie.

[26] Shim J.S., Lee J.M. (2024). Energy drink consumption among Korean adolescents: Prevalence and associated factors. Clin. Exp. Pediatr..

[27] Granda D., Surała O., Malczewska-Lenczowska J., Szczepańska B., Pastuszak A., Sarnecki R. (2024). Energy Drink Consumption Among Physically Active Polish Adolescents: Gender and Age-Specific Public Health Issue. Int. J. Public Health.

[28] Degirmenci N., Fossum I.N., Strand T.A., Vaktskjold A., Holten-Andersen M.N. (2018). Consumption of energy drinks among adolescents in Norway: A cross-sectional study. BMC Public Health.

[29] Faris M.E., Al Gharaibeh F., Islam M.R., Abdelrahim D., Saif E.R., Turki E.A., Al-Kitbi M.K., Abu-Qiyas S., Zeb F., Hasan H. (2023). Caffeinated energy drink consumption among Emirati adolescents is associated with a cluster of poor physical and mental health, and unhealthy dietary and lifestyle behaviors: A cross-sectional study. Front. Public Health.

[30] Scuri S., Petrelli F., Tesauro M., Carrozzo F., Kracmarova L., Grappasonni I. (2018). Energy drink consumption: A survey in high school students and associated psychological effects. J. Prev. Med. Hyg..

[31] Sampasa-Kanyinga H., Hamilton H.A., Chaput J.P. (2018). Sleep duration and consumption of sugar-sweetened beverages and energy drinks among adolescents. Nutrition.

