# Supplementary material for: The Frequency, Preferences, and Determinants of Energy Drink Consumption Among Young Polish People After the Introduction of the Ban on Sales to Minors
Source: Nutrients. 2025 Aug 20;17(16):2689. doi: 10.3390/nu17162689 (PMC12389082; doi:10.3390/nu17162689)
Supplement: Supplementary file 1 [file nutrients-17-02689-s001.zip › nutrients-3780642-supplementary.pdf]

# Supplementary File S1. Survey in Polish

## CZĘSTOTLIWOŚĆ, PREFERENCJE I UWARUNKOWANIA SPOŻYCIA NAPOJÓW ENERGETYCZNYCH WŚRÓD MŁODYCH POLAKÓW PO WPROWADZENIU ZAKAZU SPRZEDAŻY NIELETNIM

Jesteśmy studentkami Uniwersytetu Rzeszowskiego na kierunku dietetyka II stopnia. Należymy do Studenckiego Koła Naukowego Żywienia Człowieka. Zwracamy się z prośbą o uzupełnienie poniższego kwestionariusza dotyczącego spożycia napojów energetyzujących. Ankieta jest całkowicie anonimowa, a wyniki zostaną wykorzystane wyłącznie w celach naukowych. W każdym momencie mogą Państwo zrezygnować z badania. Bardzo dziękujmy za poświęcony czas!

*Wymagane

1. **Czy pijesz napoje energetyzujące? ***
   - ☐ TAK
   - ☐ NIE

2. **Jak często je spożywasz?**
   - ☐ Kilka razy dziennie
   - ☐ Raz dziennie
   - ☐ >1 w tygodniu
   - ☐ <1 w tygodniu
   - ☐ Bardzo rzadko

3. **Z jakiego powodu je spożywasz?**
   **(możesz wybrać kilka odpowiedzi)**
   - ☐ Dla smaku
   - ☐ Przy większym wysiłku fizycznym
   - ☐ Dla zwiększenia energii
   - ☐ Przy znacznym zmęczeniu
   - ☐ Reklamują je popularne osoby
   - ☐ Dla towarzystwa ze znajomymi
   - ☐ Wszyscy je piją

4. **Na co zwracasz uwagę przy zakupie tego typu napojów?**
   **(możesz wybrać kilka odpowiedzi)**
   - ☐ Cena
   - ☐ Smak
   - ☐ Wielkość opakowania
   - ☐ Zawartość kofeiny
   - ☐ Skład
   - ☐ Opinia znajomych
   - ☐ Nie przywiązuję uwagi do wyboru napoju

5. **Jaką wielkość napoju energetyzującego najczęściej kupujesz?**
   - ☐ 250 ml
   - ☐ 355 ml
   - ☐ 500 ml

6. **Czy od momentu wejścia w życie ZAKAZU sprzedaży "energetyków" osobom niepełnoletnim ograniczyłeś/aś spożycie tego typu napojów?**
   - ☐ TAK
   - ☐ NIE

7. **Czy zauważyłeś/ łaś, że z biegiem czasu ilość wypijanych przez ciebie energetyków zmieniła się?**
   - ☐ Tak, wzrosła
   - ☐ Tak, zmniejszyła się
   - ☐ Nie zmieniła się

8. **Czy próbowałeś/ łaś ograniczyć spożycie napojów energetyzujących?**
   - ☐ TAK
   - ☐ NIE

9. **Płeć ***
   - ☐ Kobieta
   - ☐ Mężczyzna
   - ☐ Inna

10. **Wiek ***
    - ☐ 15 lat
    - ☐ 16 lat
    - ☐ 17 lat
    - ☐ 18 lat
    - ☐ 19 lat
    - ☐ 20 lat

11. **Miejsce zamieszkania ***
    - ☐ Duże miasto (powyżej 20 tys. mieszkańców)
    - ☐ Małe miasto (poniżej 20 tys. mieszkańców)
    - ☐ Wieś

## Supplementary File S1. Survey in English

### FREQUENCY, PREFERENCES, AND DETERMINANTS OF ENERGY DRINK CONSUMPTION AMONG YOUNG POLISH PEOPLE AFTER THE INTRODUCTION OF THE BAN ON SALES TO MINORS

We are master's students in the field of Dietetics at the University of Rzeszów and members of the Student Scientific Club of Human Nutrition. We kindly ask you to complete the following questionnaire regarding the consumption of energy drinks. The survey is entirely anonymous, and the results will be used exclusively for scientific purposes. You may withdraw from the study at any time. Thank you very much for your time and participation!

* Required response

12. **Do you consume energy drinks?** *
    - ☐ YES
    - ☐ NO

13. **How often do you consume them?**
    - ☐ Several times a day
    - ☐ Once a day
    - ☐ More than once a week
    - ☐ Less than once a week
    - ☐ Very rarely

14. **What are your reasons for consuming energy drinks?**
    ***(You may select more than one answer)***
    - ☐ Because of the taste
    - ☐ As a support for physical activity
    - ☐ To increase energy
    - ☐ When very tired
    - ☐ Because of advertisements and the presence of celebrities in them
    - ☐ For company with friends
    - ☐ Because of fashion

15. **What factors influence your choice when purchasing energy drinks?**
    ***(You may select more than one answer)***
    - ☐ Price
    - ☐ Taste
    - ☐ Package size
    - ☐ Caffeine content
    - ☐ Ingredients
    - ☐ Friends' opinions
    - ☐ I do not pay attention to the choice of drink

16. **What volume of energy drink do you most frequently purchase?**
    - ☐ 250 ml
    - ☐ 355 ml
    - ☐ 500 ml

17. **Since the introduction of the legal ban on the sale of energy drinks to minors, have you reduced your consumption?**
    - ☐ YES
    - ☐ NO

18. **Have you noticed any changes over time in the amount of energy drinks you consume?**
    - ☐ Yes, it has increased
    - ☐ Yes, it has decreased
    - ☐ No, it has remained the same

19. **Have you attempted to reduce your consumption of energy drinks?**
    - ☐ YES
    - ☐ NO

20. **Sex** *
    - ☐ Woman
    - ☐ Men
    - ☐ Other

21. **Age** *
    - ☐ 15
    - ☐ 16
    - ☐ 17

22. **Place of residence** *
    - ☐ Large city (>20 thousand inhabitants)
    - ☐ Small town (<20 thousand inhabitants)
    - ☐ Village

## Supplementary File S2. Information for Parent/Legal Guardian in Polish

**Szanowni Państwo,**

uprzejmie informujemy, że Państwa dziecko zostało zaproszone do udziału w anonimowym badaniu naukowym pt.:
**„Częstotliwość, preferencje i uwarunkowania spożycia napojów energetycznych wśród młodych Polaków po wprowadzeniu zakazu sprzedaży nieletnim"**, które realizowane jest przez studentki kierunku **dietetyka II stopnia Uniwersytetu Rzeszowskiego**, działające w ramach **Studenckiego Koła Naukowego Żywienia Człowieka**.

Celem badania jest poznanie skali i uwarunkowań spożywania napojów energetyzujących wśród młodzieży szkolnej po wprowadzeniu nowych regulacji dotyczących zakazu sprzedaży tych produktów osobom niepełnoletnim.

**Uprzejmie zapewniamy, że:**

- udział w ankiecie jest **całkowicie dobrowolny i anonimowy**,
- kwestionariusz **nie zawiera żadnych danych wrażliwych ani informacji pozwalających na identyfikację uczestników**,
- **wyniki zostaną wykorzystane wyłącznie do celów naukowych i statystycznych**,
- Państwa dziecko może **odmówić udziału lub przerwać wypełnianie ankiety na każdym etapie**, bez podawania przyczyny i bez żadnych konsekwencji.

Będziemy wdzięczne za umożliwienie dziecku udziału w badaniu, które pozwoli lepiej zrozumieć współczesne nawyki zdrowotne młodzieży i posłuży do opracowania działań edukacyjnych sprzyjających ochronie zdrowia.

W przypadku pytań uprzejmie prosimy o kontakt:
**Studenckie Koło Naukowe Żywienia Człowieka, Uniwersytet Rzeszowski**
(e-mail: …………………………, opiekun naukowy:…………………………………….)

Z wyrazami szacunku,
**Zespół badawczy**
Uniwersytet Rzeszowski, kierunek Dietetyka

## Supplementary File S2. Information for Parent/Legal Guardian in English

**Dear Parent/Legal Guardian,**

We would like to inform you that your child has been invited to take part in an anonymous scientific study entitled:
**“Frequency, Preferences and Determinants of Energy Drink Consumption among Young Poles after the Introduction of a Ban on Sales to Minors”**, conducted by Master’s students of **Dietetics at the University of Rzeszów**, who are members of the **Student Scientific Circle of Human Nutrition**.

The aim of the study is to assess the frequency and factors associated with energy drink consumption among school-aged youth following the implementation of new regulations prohibiting the sale of such products to minors.

**Please note that:**

- participation in the survey is **entirely voluntary and anonymous**,
- the questionnaire **does not include any sensitive data or information that would allow identification of the participants**,
- the results will be used **solely for scientific and statistical purposes**,
- your child may **refuse to participate or withdraw from the survey at any stage**, without providing any reason and without any consequences.

We would be grateful for your support in allowing your child to participate in this study, which will help us better understand the health-related behaviors of young people and develop educational measures to promote their wellbeing.

If you have any questions, please contact:
**Student Scientific Circle of Human Nutrition, University of Rzeszów**
(email: ……………., academic supervisor: ……………………….).

With kind regards,
**Research Team**
University of Rzeszów, Dietetics
